# Illicit Online Pharmacies: A Scoping Review

**DOI:** 10.3390/ijerph20095748

**Published:** 2023-05-08

**Authors:** Yam B. Limbu, Bruce A. Huhmann

**Affiliations:** 1 Feliciano School of Business, Montclair State University, 1 Normal Ave., Montclair, NJ 07043, USA; 2 Department of Marketing, Virginia Commonwealth University, Richmond, VA 23284, USA

**Keywords:** illicit online pharmacies, prescription drug, scoping review, patient safety, pharmaceutical marketing and supply chain, public health awareness and education

## Abstract

This scoping review presents the extent and nature of the body of literature on illicit online pharmacies (IOPs) and identifies research gaps. Using the five-step framework developed by Arksey and O’Malley, we searched PubMed, Web of Science, EMBASE, CINAHL, Science Direct and PsycInfo to retrieve relevant studies published in English in peer-reviewed journals. The search strategy identified forty-three articles that met the inclusion criteria. Ten themes were identified and categorized into five clusters: patient risk, healthcare providers, marketing and supply chain, public health and society, and policy and regulation. Research into these clusters has evolved over time and has focused increasingly on issues related to specific drugs rather than the overall phenomenon. Data collection has been dominated by convenience sampling, online searches, content analysis and surveys. Data analysis remains primarily descriptive. Gaps within the extant literature suggest an agenda for future research into regulation and enforcement; public health awareness and education; healthcare services; risks to patients and public health; patient-, price- and product-related issues; website design; social media promotion; and supply chains and logistics. We conclude that IOPs are vastly understudied and suggest an urgent need for further empirical and conclusive research.

## 1. Introduction

An online pharmacy (also known as an Internet pharmacy or e-pharmacy) sells prescription and over-the-counter drugs through its website. The number of online pharmacies has grown exponentially over the last two decades with the rapid expansion of the Internet and patients’ increasing acceptance of online information for self-diagnosis and their desire to reduce healthcare costs. The market size of global online pharmacies was USD 68 billion in 2021 and is expected to reach USD 206 billion annually by 2028, with a compound annual growth rate (CAGR) of 16.8% [1]. The U.S. online pharmacy market is expected to reach USD 146 billion in 2026, with a CAGR of approximately 19% [2]. 

The substantial growth of online pharmacies has exacerbated illegal sales of medications. Online medical misinformation and illegal sales of unapproved and misbranded prescription drugs have intensified since the beginning of the COVID-19 pandemic. Much of online pharmacies’ exponential growth can be attributed to illicit online pharmacies. In this study, we define illicit online pharmacies (IOPs, also known as illegal, illegitimate or rogue online pharmacies) as websites that violate regulations by selling counterfeit, adulterated or unapproved drugs or dispensing prescription drugs without a valid prescription. IOPs do not meet national or international pharmacy regulations, nor have IOPs been subjected to the requisite regulatory review, licensure and/or certification [3]. In the U.S., IOPs violate the *Federal Food, Drug, and Cosmetic Act* (*FD&C Act*) by selling misbranded or unapproved prescription drugs of unknown origin, safety and effectiveness, as well as prescription drugs, controlled substances or other medical treatments without required warnings about associated health risks, adequate directions for safe use, or requiring valid prescriptions [4]. IOPs often offer deep discounts [5] and lower prices than those of legitimate Internet or brick-and-mortar pharmacies [6]. In addition, IOPs occasionally operate with the cooperation of a physician or group of doctors [7] who can prescribe medications without human interaction to assess patients’ needs. Frequently, IOP websites depict physicians and other medical professionals to create false impressions about the involvement of medical professionals. Thus, operating as an IOP tends not to be the result of an oversight nor a lapse of regulatory compliance. Instead, it is almost exclusively a flouting of regulatory strictures to profit from selling counterfeit, adulterated or unapproved drugs to patients seeking an alternative source of medication.

Several IOPs operate multiple websites, often from undisclosed locations, and some are controlled by organized criminal networks [8]. In fact, pharmaceutical and over-the-counter drug counterfeiting and online distribution are now the largest form of organized crime and represent USD 75 billion annually [9]. Given the relatively low cost of additional web addresses, IOPs may easily proliferate their operations across multiple websites, often hosted outside the reach of U.S. authorities, making it difficult to enforce regulations. For example, a recent study of warning letters to online pharmacies found that one-fourth referenced more than 20 websites, and one in five online pharmacies that received a warning for violating regulatory guidelines ignored the Food and Drug Administration’s compliance request [10]. 

IOPs far outnumber legal online pharmacies. Astonishingly, of the more than 35,000 online pharmacies operating worldwide, 95% violate the regulations or laws in the jurisdiction(s) where they operate or distribute medications [11]. Furthermore, a study of the first page of search engine results for purported COVID-19 treatment queries found that 78% of online pharmacies listed are IOPs, and most do not require valid prescriptions [12].

In addition to cost savings and convenience, patients purchase from IOPs to self-medicate and overcome their lack of a prescription, healthcare providers’ refusal to prescribe, dissatisfaction with providers or embarrassment regarding their condition [10]. Prescription-only medicines are widely available and easily accessible online, which increases patient safety concerns [13]. Such unrestricted access to prescription drugs online also facilitates illegal and deviant drug use and legitimizes unacceptable health practices [14]. Thus, IOPs pose serious risks to individual patients as well as to public health by distributing counterfeit, adulterated and unapproved drugs; promoting self-diagnosis and self-medication; aiding drug misuse and abuse; and encouraging medically unnecessary or overprescribing behavior [15,16]. In addition to patient and public health risks, IOPs violate professional, legal and ethical medical standards, leading to serious economic, social and psychological consequences [8].

Lately, IOPs have been a subject of interest to various stakeholders, including government regulators, public policymakers, consumer advocates, ethicists and the medical community; however, little empirical research has been published on the topic [17]. Little is known about the key risk characteristics, central challenges, and current legal, regulatory and law enforcement responses [3]. This area of investigation is still in its infancy, and the general scope of research on the topic is unavailable in the literature. In light of the explosive growth of online pharmacies, a more comprehensive understanding of IOPs is urgently needed. Therefore, the current scoping review aims to offer a broad overview of this literature, with attention primarily devoted to research themes and subthemes. More specifically, this scoping review identifies key themes arising in the IOP literature, main gaps where additional research is necessary and directions for future research.

## 2. Methods

Because our purpose was to map the extent of the literature, a scoping review was chosen, as this approach integrates findings from qualitative and quantitative studies that address similar, overlapping or complementary research questions within a single review [18,19]. A scoping review determines the scope of the literature on a topic, identifies knowledge gaps, clarifies concepts, investigates research conduct, and informs future systematic reviews [19,20,21,22]. This method is appropriate for addressing broader, more complex and exploratory research questions. Moreover, it is relevant in fields containing a paucity of rigorous evidence [20,23]. Finally, scoping reviews are useful for reviewing evidence rapidly in emerging fields or topics [22], such as the topic covered in the present study. This scoping review follows Arksey’s and O’Malley’s [20] five-step framework: (1) research question identification, (2) relevant study identification, (3) study selection, (4) data charting and investigation and (5) result presentation. 

### 2.1. Identifying Research Questions

The first step is to identify the scoping review’s research question(s). This review aims to synthesize key topics covered in the academic literature pertaining to IOPs and to identify critical knowledge gaps and future research directions. Thus, we investigate the following broad research questions.

RQ1: What are the themes of previous research on illicit online pharmacies?
RQ1a: What are the themes of previous research on illicit online pharmacies by year?
RQ1b: What are the themes of previous research on illicit online pharmacies in terms of data collection methods?
RQ2: How has previous research on illicit online pharmacies changed over time?

### 2.2. Identifying Relevant Studies

The second step concerns the scoping review’s data sources and search strategy. This scoping review was performed according to the Preferred Reporting Items for Systematic Reviews and Meta-Analyses for scoping reviews (PRISMA-ScR) [24]. To achieve ‘broad coverage’ of the available literature and retrieve a wide range of relevant articles [20], we searched six electronic databases (PubMed, Web of Science, EMBASE, CINAHL, Science Direct and PsycInfo). Table 1 presents the search terms and Boolean operators.

### 2.3. Study Selection

Study selection is the third phase in a scoping review [20]. Inclusion in the current review required publications to (1) be original qualitative and/or quantitative research, (2) focus on IOPs, (3) adequately describe research design and report results and (4) be published in English in peer-reviewed journals.

**Figure 1:**
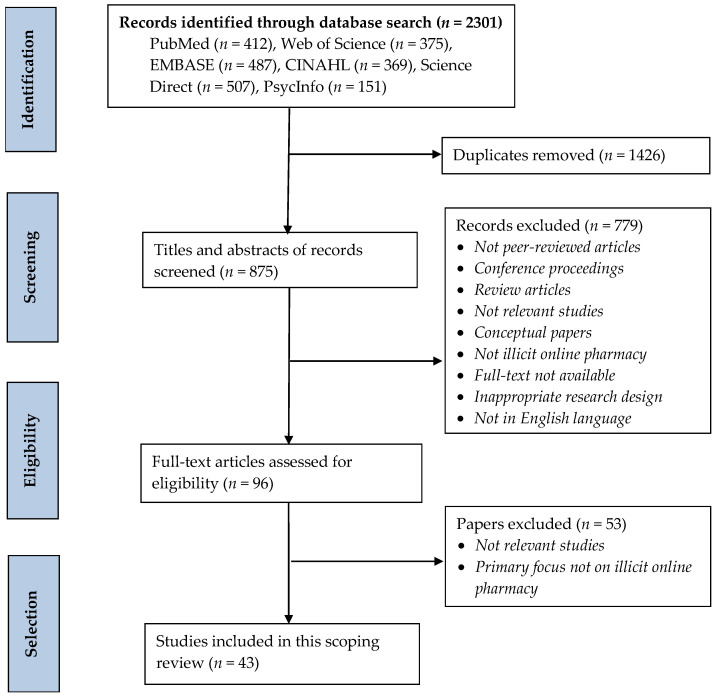
PRISMA-ScR flow diagram showing search strategy and study selection process.

As shown in Figure 1, the PRISMA-ScR flow diagram summarizes the study selection process and eligibility criteria, which involve three stages: identification, screening and eligibility. In the identification phase, the search strategy identified 2301 records. After removing duplicates, the remaining 875 records were further screened. Of these, 799 studies were excluded after screening the titles and abstracts. These studies excluded from the final analysis included conceptual articles, grey literature, narrative reviews, case studies, commentaries, non-peer-reviewed articles, conference proceedings, articles for which the full-text was unavailable, studies with inappropriate research designs and articles not written in English. The remaining 96 full-text articles were further assessed for eligibility. Of these, 53 articles were removed, as they were irrelevant or did not primarily focus on IOPs despite using relevant keywords. The remaining 43 studies were deemed eligible for this review.

### 2.4. Charting the Data

In the fourth stage of this scoping review, two researchers extracted data from the included studies independently and generated a descriptive summary of the results. The key information extracted from each study included author(s), publication year, journal name, country of publication, study objective, study design, study method, sampling unit, sample size, themes and main findings.

### 2.5. Collating, Summarizing and Reporting Results

The final step of a scoping review involves collating, summarizing and reporting results in line with the study objectives [20]. These results are reported in the following section. The data were analyzed using IBM SPSS Statistics 27.

## 3. Results

### 3.1. Characteristics of Studies Included in This Scoping Review

First, the characteristics of studies included in the review were summarized using frequencies and percentages. Examining these characteristics revealed several interesting observations about the studies. 

Although research on IOPs dates back to 2006, the body of IOP literature has been growing. No studies were published in 2007–2010 or in 2014; however, seven studies were published in 2017, followed by another wave of studies during the COVID-19 pandemic. Although IOP studies have appeared in 29 journals across various disciplines (e.g., public health, pharmaceutical science, business and medicine), almost one-fourth of the articles (23.3%) appeared in the *Journal of Medical Internet Research*. Although online retailing, in general, has received significant attention from business and marketing scholars, only one study on IOPs was published in a business journal [25].

We observed some patterns in the sources of IOP research. Authors who frequently published IOP research included Tim K. Mackey, Andras Fitter and Bryan A. Liang. Most studies (67.4%) collected data from multiple countries. Among single-country studies, 42.8% focused on the U.S. 

Finally, the results revealed some prevailing practices in the sample population, data collection procedures and data analysis of IOP research. The most common population was online pharmacy websites (47.4%). Other populations included tweets, hashtags, comments, posts, visits, general adult populations, employees and students. All but one study used a convenience sample. Thirteen studies (30.9%) collected data through online searches. Other studies often used a content analysis (23.3%) or surveys (18.6%). Only four studies were qualitative, and almost all (90.7%) were quantitative. Data collection most commonly occurred in 2015 (nine studies), followed by five studies that collected data in 2011, four in 2018 and four in 2019. Twenty-four studies reported only frequency counts and percentages. Thirty-nine were cross-sectional, and four were longitudinal studies. 

### 3.2. What Are the Themes of Previous Research on Illicit Online Pharmacies?

**Figure 2:**
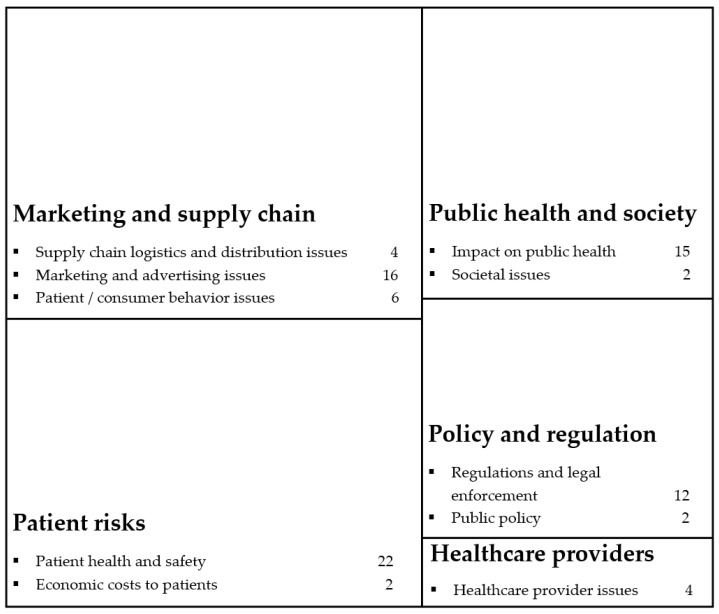
Clusters of themes covered in the IOP literature.

Identifying the themes was a recursive process of familiarizing ourselves with the studies, then generating and refining themes through multiple examinations of research questions addressed, variables measured and the focal interest of each study. Through a careful reading of the studies, ten themes emerged from this scoping review. Some studies had multiple themes. Figure 2 shows the number of studies investigating issues related to each theme. The most common theme was IOPs’ impact on patient health and safety in twenty-two studies [8,13,26,27,28,29,30,31,32,33,34,35,36,37,38,39,40,41,42,43]. Sixteen studies focused on marketing and advertising issues with IOPs [6,8,16,25,36,37,38,44,45,46,47,48,49,50,51,52]. IOPs’ impact on public health was investigated in fifteen studies [5,12,16,31,32,41,43,44,46,48,51,53,54,55,56]. Twelve studies focused on regulations and legal enforcement issues with IOPs [5,8,13,26,28,45,50,53,56,57,58,59]. Other themes included patient/consumer behavior toward IOPs [33,34,40,49,59], supply chain issues with IOPs [35,42,55,60], healthcare provider issues with IOPs [27,43,54,60], societal issues with IOPs [36,52], public policy issues with IOPs [25,30] and economic costs to patients [29,38].

As shown in Figure 2, the ten themes identified above were further categorized into five larger clusters. The first cluster represented marketing and supply chain issues. This cluster focused on IOP sales, from obtaining supplies to promoting sales to interacting with potential purchasers. Thus, its three themes dealt with IOP issues related to marketing and advertising, patient or consumer behavior, and distribution or supply chains. The second cluster, public health and society, included two themes dealing with broader harms that IOPs pose to public health and society at large. The third cluster was policy and regulation. Its two themes dealt with governmental efforts to curb IOPs through regulatory and legal enforcement or public policy. The fourth cluster, patient risk, included two themes focused on individual-level harms. These themes dealt with patient health and safety as well as economic risks associated with IOPs. Finally, the fifth cluster represented IOP-related issues among healthcare providers, such as their awareness and knowledge of IOPs, their actions to discourage patient use of IOPs and the impact of IOPs on their practice.

Again, most studies were categorized into more than one cluster of themes (also known as a thematic cluster). For example, Penley et al. [6] examined the marketing, patient safety and drug cost of insulin (both Humalog and NovoLog) sold by Internet pharmacies. Thus, their study was categorized as investigating both the marketing and supply chain and the patient risk clusters.

### 3.3. What Are the Themes of Previous Research on Illicit Online Pharmacies by Year?

**Figure 3:**
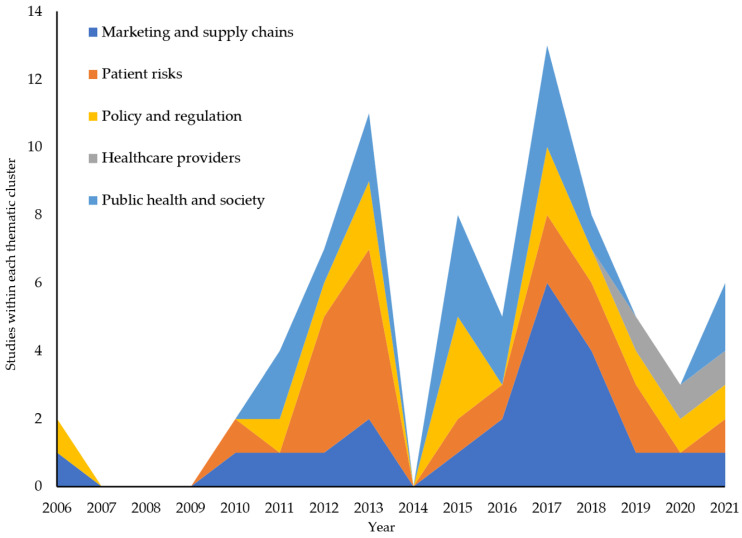
Studies within each thematic cluster by publication year.

Figure 3 depicts how IOP research into the clusters has changed over the years. After a couple of seminal papers in 2006, no research on IOPs was published for the next few years. Then, a wave that was heavily focused on patient risk peaked in 2013, followed by additional interest in public policy and regulation (peaking 2015), public health and society (peaking 2015–2017) and marketing and supply chain (peaking 2017). From 2018 to 2020, research publications on IOPs declined, although research into healthcare provider issues started to appear. Following the onset of the COVID-19 pandemic, another wave of interest in IOPs started, with growing research in the patient risk and public health and society clusters. IOP research has evolved from a disproportionate focus on a few themes to a more balanced investigation across all aspects of the phenomenon.

### 3.4. What Are the Themes of Previous Research on Illicit Online Pharmacies in Terms of Data Collection Method?

**Figure 4:**
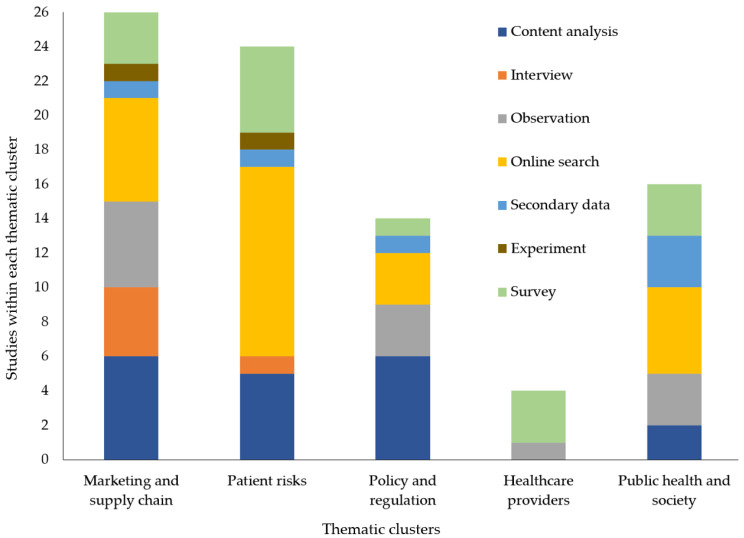
Studies within each thematic cluster by data collection method.

Figure 4 shows which data collection methods have been used to study the various thematic clusters. Pharmacy researchers, in general, tend to be more familiar with nonexperimental (e.g., observing subjects’ behavior, analyzing previously available secondary or archival data and using questionnaires to survey respondents) and various true and quasi-experimental methods rather than qualitative methods, of which interviews are most common in pharmacy research [61,62]. Research on marketing and supply chain issues has been conducted with a mix of methods, although only 4% of studies in this cluster used secondary data. Data were commonly collected for marketing and supply chain studies via an online search of search engines such as Google or Bing (24%), content analysis of information on websites or social media posts (24%) and observational techniques (20%). Patient risks have been investigated with data from all the sources except observation. The most common (47.8%) for the patient risks cluster was online search data, followed by content analysis and surveys. Content analysis (42.9%) provided more data for policy and regulation studies than did any other data collection method. Data for studies in the healthcare providers cluster came exclusively from surveys or observation. The public health and society cluster has been investigated with all data sources except interviews. Online search data (31.3%) was most common in public health and society studies. This demonstrates the potential for researchers to enhance the literature in some clusters by employing under-utilized data collection methods to allow them to address different types of research questions and an expanded set of explanatory variables and outcomes.

### 3.5. How Has Previous Research on Illicit Online Pharmacies Changed over Time?

**Figure 5:**
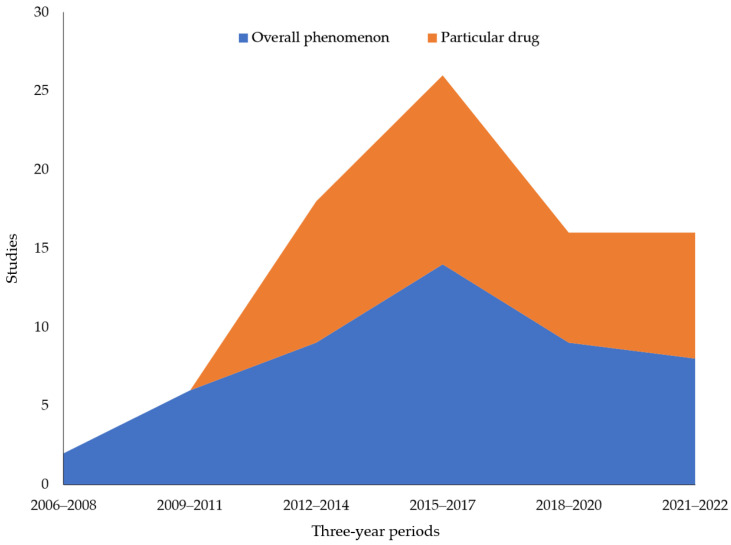
Studies that investigated a particular medication or drug category versus the overall IOP phenomenon by year of publication.

Although most research has studied the IOP phenomenon overall, some studies investigate IOPs in relation to a particular medication or drug category (e.g., fentanyl or psychiatric drugs). Drug-specific studies have become a strong component of IOP research since 2012, as shown in Figure 5. IOP studies related to a medication or therapeutic category were most common in the patient risk cluster (54.5%), followed by the marketing and supply chain cluster (46.2%). These studies tended to investigate specific harms that a medication or therapeutic category’s availability via IOPs presents to patient health. The earliest of these studies investigated erectile dysfunction drugs, contraceptives and weight-loss medications (e.g., Belviq), with more recent studies focused on insulin, oncology drugs and alleged COVID-19 treatments (e.g., dexamethasone, hydroxychloroquine or lopinavir-ritonavir). As a caveat, the comparison of drug-specific and overall IOP research depicts the year of publication, not the year of data collection. Due to the slower review process and publication speed of some journals, more studies related to IOPs offering COVID-19 treatments may be forthcoming.

As shown in Figure 5, most IOP research was not limited to a particular drug or category. Studies of IOPs, in general, were most common in the healthcare provider (75%), public health and society (68.7%) and policy and regulation clusters (57.1%). In addition, these studies of the overall phenomenon tended to investigate efforts to combat the problem of IOPs and their overall harms. Both overall studies and those focused on particular drugs or categories peaked in the 2015–2017 period.

**Figure 6:**
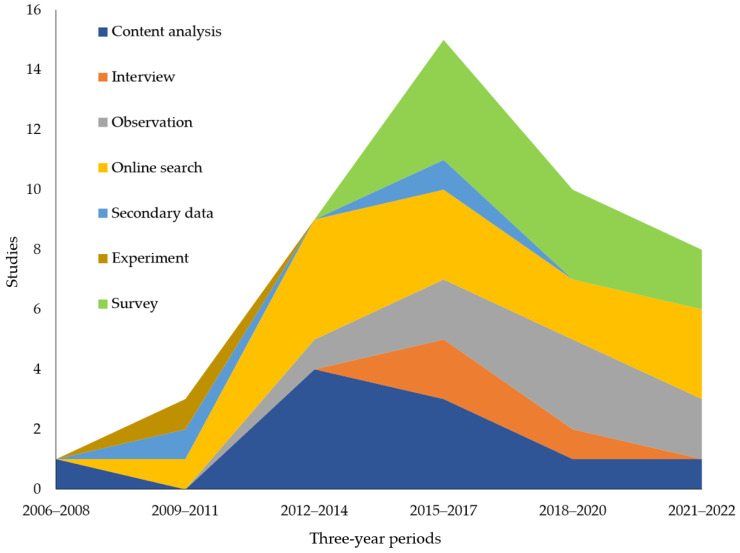
Data collection method in IOP research by year of publication.

Figure 6 shows trends in the methods used for data collection over time. The initial IOP research was based on data collected through content analyses of IOP websites. Studies based on experimental, online search and secondary data began in the 2009–2011 period. The use of content analysis and online search data grew during the 2012–2014 period and was joined by studies relying on observational data. Studies using secondary, interview and survey data joined these data collection methods in the 2015–2017 period. Although data continued to be collected via a variety of methods, no studies used interviews in the 2021–2022 period nor secondary data in either the 2018–2020 or 2021–2022 periods. This may indicate the potential to conduct interviews or secondary data research to investigate research questions related to events that have occurred in recent years, such as those related to online pharmacies in the post-pandemic period.

**Figure 7:**
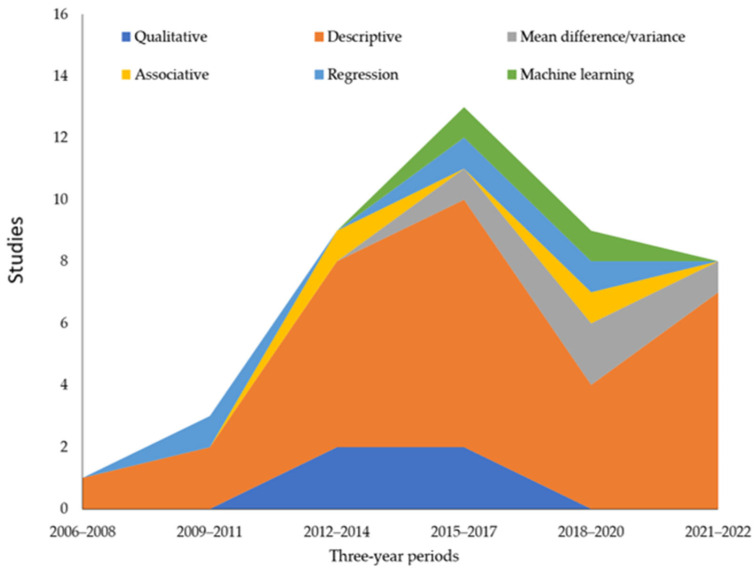
Data analysis in IOP research over time.

As shown in Figure 7, descriptive analysis (e.g., frequency counts and percentages) remains the most common form of data analysis. Further examination reveals that descriptive analysis has been applied in most studies in the public health and society cluster (81.3% of studies in this cluster), healthcare providers cluster (76%), patient risks cluster (66.6%), marketing and supply chain cluster (57.7%) and policy and regulation cluster (50%).

Other forms of data analysis have been becoming more common over the past decade. Qualitative research only appeared in the 2012–2014 and 2015–2017 periods, most often in the policy and regulation (21% of studies in this cluster), patient risks (8.3%) and marketing and supply chain clusters (7.7%). Studies analyzing mean differences or variances between groups began in 2015–2017 and have appeared most often in the patient risks (16.7% of studies in this cluster) and the marketing and supply chain clusters (11.5%). Although still less common than descriptive analysis, forms of mean difference/variance analyses have been incrementally applied more often over the past decade. Associative analysis, regression analysis and machine learning remain rare. Machine learning has been used to evaluate large corpora of social media posts in the IOP literature. For example, one study analyzed 213,041 tweets for opioid sales and found 15 that promoted IOPs, 11 posted by individual sellers of opioids, and 7 for marketing affiliates directing purchasers to other vendors [45]. 

## 4. General Discussion

With IOPs’ exponential growth, sales of misbranded, counterfeit, adulterated or unapproved medications have intensified to pose serious risks to individual and public health. Further, IOPs violate professional, legal and ethical standards with serious economic, social and psychological consequences (e.g., [8]). Thus, a more comprehensive understanding of the literature on the topic is urgently needed. The current study summarizes the nature of the extant literature on this vital topic, which has yet to be examined by any scoping or systematic reviews to the best of the authors’ knowledge. Our findings provide important insights into the state of research on IOPs and help to gain a deeper holistic understanding of this multidisciplinary literature.

We next offer directions for future research efforts based on our scoping review of the IOP literature. The gaps we identify are specifically focused on addressing the most pressing issues associated with illegitimate pharmacies.

## 5. Directions for Future Research

From a methodological perspective, our findings show that most studies collected data by using online searches and content analysis. Such data are useful to identify and describe the nature of the problem. In the future, however, researchers should consider other approaches, including surveys and experiments, to begin investigating causes and testing possible interventions to reduce IOP usage by the public. Surveys and experiments can also test the effectiveness of public health education materials and campaigns. Public health education may be a key strategy to combat IOPs. Legitimate pharmacies and health insurance providers also have incentives to educate the public regarding the dangers of IOPs, as legitimate pharmacies may lose business to IOPs, and insurance providers could reduce expensive negative health consequences from ineffective treatments or dangerous substances.

Further, this scoping review also shows that only a handful of studies on the topic were longitudinal. Future studies with longitudinal designs should examine trends in the nature and usage of IOPs as well as the long-term impacts of IOPs on patients and public health. 

Most IOP studies have been exploratory in nature, reporting only frequency counts and percentages. Future research should apply conclusive research designs. We recommend that future research adopt predictive analytics approaches and investigate causal inferences. 

According to our scoping review of IOP studies, the most common population was online pharmacy websites. In the future, researchers should expand their focus to more samples drawn from patients and healthcare providers. Moreover, future research should employ random sampling techniques rather than the convenience samples pervasive in the prior literature to enhance generalizability.

This scoping review indicates that research themes primarily focused on IOPs’ impact on patient health and safety, marketing and advertising issues with IOPs, IOPs’ impact on public health and regulatory and legal enforcement issues with IOP. Additional research is needed to expand knowledge on the themes of patient/consumer behavior toward IOPs, supply chain issues with IOPs, healthcare provider issues with IOPs, societal issues with IOPs, public policy issues with IOPs and IOPs’ economic costs to patients. 

Surprisingly, the studies included in this scoping review rarely applied or tested theories, indicating that prior research was in the early stages of identifying and describing the phenomenon rather than making and testing predictions. Nevertheless, as IOP research matures, some relevant sources of theory may be drawn from fields such as health communication, psychology, information systems, consumer behavior, sociology, retailing and economics. These theories should be used to predict or explain patient behavior; the impact of regulatory changes, public health interventions or education campaigns; and how shifts in macro-environmental influences affect IOP prevalence and usage.

Our scoping review shows that IOP research is still in its infancy. The authors conclude that IOPs are vastly understudied and suggest an urgent need for further empirical and conclusive research. Hence, we offer several avenues for future research (see Table 2).

## 6. Conclusions

This scoping review evaluates the breadth and depth of the illicit online pharmacy literature. Thus, this review should interest the many parties concerned with ending IOPs’ role in drug distribution. Public policymakers, healthcare providers, researchers interested in healthcare and ethics, insurers, law enforcement, non-governmental organizations and consumer advocates should be more aware of the scope of research on this practice. This review should help them better understand IOPs and the work required to assure patient access to prescription drugs through safe and legitimate pharmacies and to reduce opportunities for IOPs to distribute drugs online. The disruption of IOPs as a source of counterfeit and unregulated drugs should reduce opportunities for unsuspecting consumers to be preyed upon by criminal organizations and dishonest foreign actors over the Internet.

This investigation is the first systematic effort to review the nature of the available literature on IOPs. It also identifies research gaps and offers a wide range of topics for future research. This scoping review identifies five streams of research in the IOP literature: patient risk, healthcare providers, marketing and supply chains, public health and society, and policy and regulation. In addition, more specific themes emerge within each of these broad clusters. Overall, the findings reveal that IOPs are an understudied area that need serious consideration from scholars of various disciplines. However, future research should focus on empirical research, which is relatively sparse in the current literature.

## Figures and Tables

**Table 1:** Search terms with Boolean operators used for search.

Search	Search Terms (Boolean Operators)
1	“illicit” OR “illegitimate” OR “illegal” OR “rogue” AND “online” AND “pharmac*”
2	“illicit” OR “illegitimate” OR “illegal” OR “rogue” AND “internet” AND “pharmac*”
3	“illicit” OR “illegitimate” OR “illegal” OR “rogue” AND “cyber” AND“pharmac*”
4	“illicit” OR “illegitimate” OR “illegal” OR “rogue” AND “e-pharmac*”

**Table 2:** Agendas for future research by topic.

Area of Investigation	Description
Regulation and enforcement	Which regulatory and enforcement actions most effectively combat IOPs or encourage greater compliance behavior? What enforcement or regulatory changes would best address challenges associated with IOPs? What factors distinguish IOPs that will versus will not seek compliance if subjected to enforcement actions? How can regulatory or enforcement regimes better use these factors to encourage compliance? What regulatory violations or forms of noncompliance are most common (e.g., counterfeiting, unapproved formulation, labeling, drug packaging or package inserts)? To what extent are accreditation and pharmacy licenses leading cross-border operators to become IOPs? How could these barriers of entry be adjusted to encourage more legitimate competition while protecting public health? Are some nations’ regulatory or enforcement agencies better at curbing patient usage of IOPs? If so, why? What are the most significant obstacles to global enforcement? How can these be addressed to help nations better coordinate efforts to combat IOPs? To what extent do IOPs provide drug information required by law, such as usage information or side effects and contraindications? How does this required information’s presence or absence affect the likelihood of enforcement actions? What ethical issues do IOPs or enforcement actions and public health responses to IOPs raise? How do regulatory guidelines effectively address these ethical issues? What is the professional or ethical obligation to ensure access that regulatory or enforcement actions may interrupt?
Public health awareness and education	What initiatives and interventions have been designed to communicate and educate the public about IOPs? How effective are they? Why? What challenges do patients have in detecting illicit versus legitimate online pharmacies? How can public health officials improve patients’ accurate identification of IOPs? What message strategies and techniques most effectively raise public awareness of and educate the public about IOPs and the dangers associated with purchasing drugs from IOPs? How should theoretical frameworks developed in communication, marketing or psychology be applied to develop effective campaigns and interventions to raise awareness of and educate the public about the dangers of IOPs? What stakeholders, other than patients or regulatory and enforcement agencies, could play a role or have an ethical obligation to help combat IOPs? How should public awareness and education campaigns best persuade these stakeholders, such as healthcare providers, credit card companies, Internet service providers, search engines and drug manufacturers and suppliers?
Healthcare service	How do IOPs impact the delivery of healthcare services by hospitals, physicians and pharmacists? Are the impacts of IOPs on delivering healthcare services or quality of care greater for vulnerable populations? To what extent does access to IOPs encourage patients to abandon legitimate healthcare service providers versus supplement their healthcare services with additional purchases from IOPs? Do IOPs increase access to healthcare services among those otherwise unable to access them?
Risks to patients and public health	What are the impacts of products sold by IOPs on patient health and well-being? How do IOPs influence patients’ quality of care? How do IOPs affect public health and global access to effective pharmaceutical treatments? What role do IOPs play in drug abuse and self-medication? What are the privacy and security risks associated with IOPs for patients? What steps can be taken to better protect patients’ information? What is the extent of IOPs’ negative social and personal economic consequences, such as consumer fraud, invasion of privacy and the misuse or sale of personal information?
Patients	Who purchases drugs from IOPs in terms of benefits sought, search behavior, preferences, personality, demographics and usage? Based on the characteristics of frequent purchasers, how can public health officials better craft messages and other interventions? What are patients’ attitudes toward IOPs and the sale of pharmaceutical drugs without prescriptions? How do interventions change these attitudes? How do patients’ level of education, literacy and awareness of regulations influence their willingness to purchase from IOPs? What patient segments are at higher risk for purchasing drugs from IOPs? Does direct-to-consumer advertising encourage patients to purchase from IOPs? How do environmental influences, pricing, website design factors or sales promotions (e.g., discounts, coupons and customized offers) affect patient purchases from IOPs? How do social norms and perceived social pressure influence the likelihood of purchasing from IOPs? What theoretical frameworks from psychology, consumer research or sociology could be applied to understand patients’ IOP attitudes and behaviors?
Price	Do IOPs offer drug prices that are substantially lower than those of legitimate online pharmacies? If so, how are they able to do so? How transparent are drug prices prior to purchasing from IOPs versus legitimate pharmacies?
Product	What is the nature and quality of the unapproved or counterfeit drugs sold by IOPs? Which prescription drugs and brands tend to be sold by IOPs without a prescription? Are most or only a few drugs offered by IOPs counterfeited, adulterated or unapproved? What ethical implications arise from product-related IOP issues (e.g., unapproved and misbranded prescription drugs, drug sales without prescriptions, adequate directions for safe use or warnings about serious health risks)? How do affordability and availability issues complicate these ethical implications?
Website design	How do IOPs encourage patients to order? How are patient perceptions, attitudes, usage, and purchase behavior affected by IOP websites’ hedonic aspects, perceived usefulness, ease of use, visual sophistication, resemblance to reputable legitimate pharmacy websites, or signals of trustworthiness (e.g., third-party logos, return policies, purported endorsements of healthcare professionals or other trust cues)? What differences in website features (e.g., shopping carts, free shipping, testimonials or security seals) distinguish legitimate online pharmacies from IOPs? To what extent do IOP websites provide complete contact information? How does the presence or absence of contact information affect patient attitudes toward an IOP or patient behaviors (e.g., the extent of search or purchase)? To what extent do IOP websites include disclosures of policies, such as the terms and conditions of sale, return policy, refund policy, privacy protection policy, website security policy and information collection policy? How does the presence or absence of these policies affect patient attitudes toward the IOP or behaviors? How does including certification seals on IOP websites influence patient attitudes and behaviors? How can theoretical frameworks developed in information systems, communication, marketing or psychology be applied to explain and counteract effective IOP website design?
Social media promotion	How has the advent of social media and mobile technology impacted IOPs? How are counterfeit and unapproved drugs or controlled substances trafficked online through social media platforms? How does the social media promotion of IOPs differ across platforms (e.g., TikTok, Facebook or Twitter)? How are social media influencers and live streamers changing the promotion of IOPs?
Supply chains and logistics	How have changing supply chains affected IOPs? What is the origin and nature of the supply chain or distribution network of counterfeit and grey market drugs offered by IOPs? What are the roles of drug distributors and retailers in the growth of IOPs? How reliable are IOPs for order fulfillment? What do patients receive when orders are fulfilled (e.g., ordered drugs, counterfeits, adulterated drugs, inert substances or opioids)? How do barriers to entry designed to limit IOPs (e.g., accreditation and pharmacy licenses) increase the complexity and cost of the pharmaceutical supply chain? How does this increased complexity and cost negatively affect legitimate pharmacies and patients? To what degree could blockchain technology validate the pharmaceutical supply chain and reduce purchases of counterfeit drugs via IOPs?

## Data Availability

The data generated from this paper are available from the first author upon request.

## References

[1] Global Market Insights (2022). E-Pharmacy Market Size by Product Type (OTC Products, Prescription Medicine), Industry Analysis Report, Regional Outlook, Growth Potential, COVID-19 Impact Analysis, Competitive Market Share & Forecast, 2022–2028. https://www.gminsights.com/industry-analysis/e-pharmacy-market.

[2] Arizton Advisory & Intelligence (2021). Online Pharmacy Market—Industry Outlook & Forecast 2021–2026. https://www.arizton.com/market-reports/united-states-online-pharmacy-market.

[3] Mackey T.K., Nayyar G. (2016). Digital danger: A review of the global public health, patient safety and cybersecurity threats posed by illicit online pharmacies. Br. Med. Bull..

[4] Food and Drug Administration (2022). Internet Pharmacy Warning Letters. https://www.fda.gov/drugs/drug-supply-chain-integrity/internet-pharmacy-warning-letters.

[5] Leontiadis N., Hutchings A. (2015). Scripting the crime commission process in the illicit online prescription drug trade. J. Cybersecur..

[6] Penley B., Minshew L., Chen H.H., Eckel S., Ozawa S. (2022). Accessibility of low-cost insulin from illegitimate internet pharmacies: Cross-sectional study. J. Med. Internet Res..

[7] Dekker A.H. (2007). What is being done to address the new drug epidemic?. J. Osteopath. Med..

[8] Fittler A., Bosze G., Botz L. (2013). Evaluating aspects of online medication safety in long-term follow-up of 136 internet pharmacies: Illegal rogue online pharmacies flourish and are long-lived. J. Med. Internet Res..

[9] Peltier-Rivest D., Pacini C. (2019). Detecting counterfeit pharmaceutical drugs: A multi-stakeholder forensic accounting strategy. J. Financ. Crime.

[10] Limbu Y.B., Huhmann B.A. (2023). Online but Unlawful Sales of Unapproved and Misbranded Prescription Drugs: Internet Pharmacy Compliance with Food and Drug Administration Warning Letters. J. Consum. Aff..

[11] Alliance for Safe Online Pharmacies (2017). Online Pharmacy Behavior Perception Survey Results. https://buysaferx.pharmacy/public-awareness-campaigns/drug-importation/factsheets/online-pharmacy-consumer-behavior-and-perception-survey/.

[12] Fittler A., Adeniye L., Katz Z., Bella R. (2021). Effect of infodemic regarding the illegal sale of medications on the Internet: Evaluation of demand and online availability of ivermectin during the COVID-19 pandemic. Int. J. Environ. Res. Public Health.

[13] Vida R.G., Fittler A., Mikulka I., Ábrahám E., Sándor V., Kilár F., Botz L. (2017). Availability and quality of illegitimate somatropin products obtained from the Internet. Int. J. Clin. Pharm..

[14] Fincham J.E. (2021). Negative consequences of the widespread and inappropriate easy access to purchasing prescription medications on the Internet. Am. Health Drug Benefits.

[15] Orizio G., Merla A., Schulz P.J., Gelatti U. (2011). Quality of online pharmacies and websites selling prescription drugs: A systematic review. J. Med. Internet Res..

[16] Mackey T.K., Liang B.A. (2013). Global reach of direct-to-consumer advertising using social media for illicit online drug sales. J. Med. Internet Res..

[17] Limbu Y.B., Huhmann B.A. (2022). Ethical issues in pharmaceutical marketing: A systematic review and future research agenda. J. Glob. Mark..

[18] Harden A. (2010). Mixed-methods systematic reviews: Integrating quantitative and qualitative findings. Focus.

[19] Peters M.D.J., Godfrey C.M., Khalil H., McInerney P., Parker D., Soares C.B. (2015). Guidance for Conducting Systematic Scoping Reviews. Int. J. Evid. Based Healthc..

[20] Arksey H., O’Malley L. (2005). Scoping Studies: Towards a Methodological Framework. Int. J. Soc. Res. Methodol..

[21] Khalil H., Peters M., Godfrey C.M., McInerney P., Soares C.B., Parker D. (2016). An evidence-based approach to scoping reviews. Worldviews Evid. Based Nurs..

[22] Munn Z., Peters M.D., Stern C., Tufanaru C., McArthur A., Aromataris E. (2018). Systematic review or scoping review? Guidance for authors when choosing between a systematic or scoping review approach. BMC Med. Res. Methodol..

[23] Levac D., Colquhoun H., O’Brien K. (2010). Scoping studies: Advancing the methodology. Implement. Sci..

[24] Tricco A.C., Lillie E., Zarin W., O’Brien K.K., Colquhoun H., Levac D., Moher D., Peters M., Horsley T., Weeks L. (2018). PRISMA extension for scoping reviews (PRISMA-ScR): Checklist and explanation. Ann. Intern. Med..

[25] Forman R.F., Block L.G. (2006). The marketing of opioid medications without prescription over the Internet. J. Public Policy Mark..

[26] Liang B.A., Mackey T.K., Archer-Hayes A.N., Shinn L.M. (2013). Illicit online marketing of lorcaserin before DEA scheduling. Obesity.

[27] Ashames A., Bhandare R., AlAbdin S.Z., Alhalabi T., Jassem F. (2019). Public perception toward e-commerce of medicines and comparative pharmaceutical quality assessment study of two different products of furosemide tablets from community and illicit online pharmacies. J. Pharm. Bioallied Sci..

[28] Liang B.A., Mackey T.K., Lovett K.M. (2012). Suspect online sellers and contraceptive access. Contraception.

[29] Bachhuber M.A., Cunningham C.O. (2013). Availability of buprenorphine on the Internet for purchase without a prescription. Drug Alcohol Depend..

[30] Mackey T.K., Aung P., Liang B.A. (2015). Illicit Internet availability of drugs subject to recall and patient safety consequences. Int. J. Clin. Pharm..

[31] Liang B.A., Mackey T.K., Lovett K.M. (2013). Illegal “no prescription” internet access to narrow therapeutic index drugs. Clin. Ther..

[32] Liang B.A., Mackey T.K. (2012). Vaccine shortages and suspect online pharmacy sellers. Vaccine.

[33] Koenraadt R., van de Ven K. (2018). The Internet and lifestyle drugs: An analysis of demographic characteristics, methods, and motives of online purchasers of illicit lifestyle drugs in the Netherlands. Drugs Educ. Prev. Policy.

[34] Fittler A., Vida R.G., Káplár M., Botz L. (2018). Consumers turning to the internet pharmacy market: Cross-sectional study on the frequency and attitudes of Hungarian patients purchasing medications online. J. Med. Internet Res..

[35] Gaudiano M.C., Manna L., Rodomonte A.L., Bartolomei M., Bertocchi P., Gallinella B., Antoniella E., Muleri N., Civitelli G., Alimonti S. (2012). A survey on illegal and counterfeit medicines for the treatment of erectile dysfunctions in Italy. J. Sex. Med..

[36] Monteith S., Glenn T., Bauer R., Conell J., Bauer M. (2016). Availability of prescription drugs for bipolar disorder at online pharmacies. J. Affect. Disord..

[37] Ozawa S., Billings J., Sun Y., Yu S., Penley B. (2022). COVID-19 Treatments Sold Online Without Prescription Requirements in the United States: Cross-sectional Study Evaluating Availability, Safety and Marketing of Medications. J. Med. Internet Res..

[38] Sun Y., Hendrix A., Muluneh B., Ozawa S. (2022). Online Pharmacy Accessibility of Imatinib, An Oral Chemotherapy Medication. J. Natl. Compr. Cancer Netw..

[39] Campbell N., Clark J.P., Stecher V.J., Goldstein I. (2012). Internet-ordered Viagra (sildenafil citrate) is rarely genuine. J. Sex. Med..

[40] Ivanitskaya L., Brookins-Fisher J., O’Boyle I., Vibbert D., Erofeev D., Fulton L. (2010). Dirt cheap and without prescription: How susceptible are young US consumers to purchasing drugs from rogue internet pharmacies?. J. Med. Internet Res..

[41] Abanmy N. (2017). The extent of use of online pharmacies in Saudi Arabia. Saudi Pharm. J..

[42] Fittler A., Vida R.G., Rádics V., Botz L. (2018). A challenge for healthcare but just another opportunity for illegitimate online sellers: Dubious market of shortage oncology drugs. PLoS ONE.

[43] Hertig J.B., Kennedy T.M. (2022). Pharmacy Student Perceptions and Knowledge of Online Pharmacy Use. Am. J. Pharm. Educ..

[44] Mackey T.K., Kalyanam J. (2017). Detection of illicit online sales of fentanyls via Twitter. F1000Research.

[45] Mackey T., Kalyanam J., Klugman J., Kuzmenko E., Gupta R. (2018). Solution to detect, classify, and report illicit online marketing and sales of controlled substances via twitter: Using machine learning and web forensics to combat digital opioid access. J. Med. Internet Res..

[46] Anderson A.C., Mackey T.K., Attaran A., Liang B.A. (2016). Mapping of health communication and education strategies addressing the public health dangers of illicit online pharmacies. J. Health Commun..

[47] Shah N., Li J., Mackey T.K. (2022). An unsupervised machine learning approach for the detection and characterization of illicit drug-dealing comments and interactions on Instagram. Subst. Abus..

[48] Tyrawski J., De Andrea D.C. (2015). Pharmaceutical companies and their drugs on social media: A content analysis of drug information on popular social media sites. J. Med. Internet Res..

[49] Van de Ven K., Koenraadt R. (2017). Exploring the relationship between online buyers and sellers of image and performance enhancing drugs (IPEDs): Quality issues, trust and self-regulation. Int. J. Drug Policy.

[50] Penley B., Chen H.H., Eckel S.F., Ozawa S. (2021). Characteristics of online pharmacies selling Adderall. J. Am. Pharm. Assoc..

[51] Mackey T.K., Kalyanam J., Katsuki T., Lanckriet G. (2017). Twitter-based detection of illegal online sale of prescription opioid. Am. J. Public Health.

[52] Monteith S., Glenn T. (2018). Searching online to buy commonly prescribed psychiatric drugs. Psychiatry Res..

[53] Katsuki T., Mackey T.K., Cuomo R. (2015). Establishing a link between prescription drug abuse and illicit online pharmacies: Analysis of Twitter data. J. Med. Internet Res..

[54] Hertig J.B., James S.M., Hummel C.J., Rubin M.J. (2021). Evaluation of pharmacists’ awareness of illegal online pharmacies and perceived impact on safe access to medicines. Med. Access@ Point Care.

[55] Jena A.B., Goldman D.P. (2011). Growing Internet use may help explain the rise in prescription drug abuse in the United States. Health Aff..

[56] Liang B.A., Mackey T.K. (2011). Prevalence and global health implications of social media in direct-to-consumer drug advertising. J. Med. Internet Res..

[57] Zhao H., Muthupandi S., Kumara S. (2020). Managing illicit online pharmacies: Web analytics and predictive models study. J. Med. Internet Res..

[58] Li J., Xu Q., Shah N., Mackey T.K. (2019). A machine learning approach for the detection and characterization of illicit drug dealers on Instagram: Model evaluation study. J. Med. Internet Res..

[59] Kennedy J.P., Wilson J.M. (2017). Clicking into harm’s way: The decision to purchase regulated goods online. Am. Behav. Sci..

[60] Gong Y., Jiang N., Chen Z., Wang J., Zhang J., Feng J., Lu Z., Yin X. (2020). Over-the-counter antibiotic sales in community and online pharmacies, China. Bull. World Health Organ..

[61] Hussain R., Hassali M.A., Babar Z.-U.-D. (2019). Quantitative methods in pharmacy practice research. Encycl. Pharm. Pract. Clin. Pharm..

[62] Kaae S., Traulsen J.M. (2015). Qualitative methods in pharmacy practice research. Pharm. Pract. Res. Methods.

